# Parathyroid Adenoma with Prominent Lymphocytic Infiltrate

**DOI:** 10.1155/2015/705843

**Published:** 2015-02-25

**Authors:** Alexandros Iliadis, Triantafyllia Koletsa, Ioannis Kostopoulos, Georgia Karayannopoulou

**Affiliations:** Department of Pathology, Faculty of Medicine, Aristotle University of Thessaloniki, 54124 Thessaloniki, Greece

## Abstract

Only very few previously reported cases of pronounced lymphocytic infiltration in parathyroid adenoma can be found in the English medical literature. The objective of this report is to present such a rare case and to investigate to a certain extent the immunohistochemical profile of this rare histologic observation. The lymphoid cell population within the tumour was composed of nodule-forming B-cells and different subsets of infiltrating T-cells and caused minimal destruction of neoplastic tissue.

## 1. Introduction

Inflammatory disorders of the parathyroid gland (normal and neoplastic) are rare as compared to those of other endocrine organs. Parathyroid adenoma (PA) with marked lymphocytic infiltration with or without the destruction of tumour tissue is even rarer, with only few cases reported in the English scientific literature [1]. The total number of these cases is not related to any synchronous autoimmune disease but only primary hyperparathyroidism. We are herein presenting a case of a single orthotopic PA, which showed pronounced lymphocytic infiltration throughout, in a patient with primary hyperparathyroidism. The immunophenotype of the lymphocytic infiltration is emphasized.

## 2. Case Report

A 52-year-old female diagnosed with primary hyperparathyroidism underwent surgery for the resection of an enlarged parathyroid gland located at the lower pole of the right thyroid gland lobe. The other glands were normal and the patient was cured. No associated diseases such as generalized inflammatory conditions were clinically reported. There was no evidence of presence of autoimmune disease clinically or serologically.

A 1.9 × 1 × 0.6 cm sized, oval-shaped tissue sample, weighing at 0.6 g, showed the usual appearance of an enlarged parathyroid gland without any striking macroscopic features: a well circumscribed, tan nodule with a delicate capsule.

**Figure 1:**
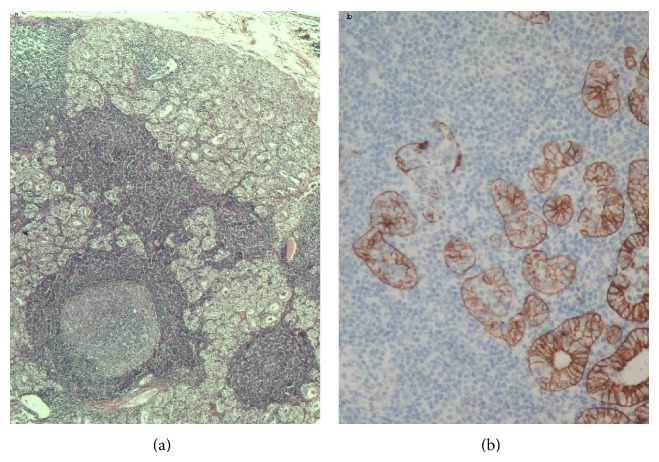
Encapsulated homogenous lesion, composed of clear type chief cells of a microfollicular pattern in delicate capillary network, accompanied by multiple, scattered foci of dense lymphocytic infiltrates with formation of follicles with fully developed germinal centers ((a): HE ×100). Small foci with glandular destruction due to lymphocytic infiltration highlighted by staining with CK8/18 ((b): IHC ×200).

Multiple sectioning revealed a neoplasm with the texture of a parathyroid gland, which exhibited hyperplasia mainly of clear type chief cells with an amphophilic cytoplasm, arranged predominantly in a microfollicular pattern and was surrounded by a thin fibrous capsule of connective tissue, without capsular or vascular invasion. At least two areas seemed to maintain strips of normal parathyroid tissue peripherally without the presence of inflammatory cells. Interestingly, amongst the tumour cell nests, which showed a positive immunostain for parathormone (PTH) (mouse mAb, clone NCL-PTH-488, 1:50, Novocastra, Newcastle, UK), there were multiple, scattered foci of dense lymphocytic infiltrates (Figure 1(a)), which did not seem to cause destruction of the surrounding parenchymatous neoplastic tissue, apart from a few small foci as demonstrated with the immunostains for CK8/18 (mouse mAb, clone 5D3, 1:50, Novocastra, Newcastle, UK) (Figure 1(b)) and CD8 (mouse mAb, clone C8/144B, 1:70, Dako, Glostrup, Denmark). In many areas the lymphocytes swarmed to the formation of follicles with fully developed germinal centres, demonstrating a mixed cellular composition in the immunostains for B- and T-cell markers, namely, CD20 (mouse mAb, clone L-26, 1:300, Dako, Glostrup, Denmark) and CD3 (mouse mAb, clone NCL-CD3-PS1, 1:30, Novocastra, Newcastle, UK). A few T-cells that coexpressed CD8 and TIA-1 (mouse mAb, clone TIA-1, 1:100, Biocare, Concord, CA, USA) antigens infiltrated the microfollicles of the neoplasm. Immunostaining for CD4 (mouse mAb, clone NCL-CD4-1F6, 1:50, Novocastra, Newcastle, UK) showed positive epithelial cells and many T4 cells, mainly around the lymph follicles, without being intraepithelial. A significant number of plasma cells were also present, which showed polytypic light chain expression. Signs of fibrosis were not to be seen. Immunostain for EBV latent membrane protein (LMP1) (mouse mAb, clone CS.1-4, 1:50, Dako, Glostrup, Denmark) was negative. The case was diagnosed as a solitary orthotopic PA, associated with prominent lymphocytic infiltration.

## 3. Discussion

The lymphocytic infiltrate in PA and hyperplastic or normal parathyroid gland is an unusual histologic observation. Its presence is not likely to imply an autoimmune disorder. The main hypothesis is that the lesion may be a result of local tissue response [2]. Another study suggested that the histological picture is consistent with an autoimmune process directed against the adenomas, indicating that this reaction had, in part, been successful in reducing the abnormal cell population [3]. In this case, there was evidence of the immune response effort to destroy follicles, but this phenomenon was limited to some foci, without significant morphological or at least functional effect on the adenoma, since hyperparathyroidism was present. Hence, similar cases should be considered as an immunoresponse to the adenoma and this concept is reinforced by the fact that there was no inflammatory infiltrate in the adjacent rim of the remnant parathyroid gland.

In this context, the absence of lymphocytic infiltration in the remnant parathyroid gland strongly suggests that the possibility of a preadenoma lymphocytic parathyroiditis is quite implausible. The term parathyroiditis has been used inconsistently and has neither an agreed classification scheme nor a steady clinical association. It seems that the histological evidence of inflammation within the parathyroids has never been shown to be the definitive pendant of autoimmune hypoparathyroidism or any other parathyroid dysfunction for that matter [4]. As opposed to lymphocytic infiltrations of the parathyroid combined with underlying systemic diseases, such as septicaemia and myocardial infarction, that are not that uncommon as shown by autopsy studies, genuine parathyroiditis as a primary, organ-specific immune process is a rare condition. Current data cannot allow plausible explanations with regard to its origin. Arguably, gland hyperfunction and cell hyperplasia must have something to do with it, insofar that in certain microenvironments they become triggers for an immunoresponse aiming at suppressing the hyperfunction, albeit unsuccessfully.

In their review study on inflammatory diseases of the parathyroid gland, Talat et al. also included cases of adenomas with inflammatory infiltration and suggested two different infiltration patterns. Our case seems to best fit the description for the specific pattern that reads “*the pattern of lymphocytic parathyroiditis is characterized by interstitial lymphocytes away from the vessels with terminal differentiation* (*plasma cells*)* and/or formation of germinal centres*,” in contrast to the nonspecific pattern “*marked by diffuse lymphocytic infiltrates in the direct vicinity of venules without evidence of lymphocyte maturation or immune-mediated tissue damage*” [4].

The nature of the lymphoid infiltrate was analyzed to hopefully unveil something more as regards the pathogenesis of this process. This cell population reflects an organized immune process and is composed of both infiltrating T-cells and compact nodule-forming B-cells. We tend to agree with the hypothesis that lymphocytic infiltration in parathyroid adenomas is a mysterious but clinically innocuous histological finding and its presence does not imply an autoimmune disorder [5].
